# Mitochondrial Targeting Therapeutics: Promising Role of Natural Products in Non-alcoholic Fatty Liver Disease

**DOI:** 10.3389/fphar.2021.796207

**Published:** 2021-12-23

**Authors:** Jingqi Xu, Jiayan Shen, Ruolan Yuan, Bona Jia, Yiwen Zhang, Sijian Wang, Yi Zhang, Mengyang Liu, Tao Wang

**Affiliations:** ^1^ State Key Laboratory of Component-based Chinese Medicine, Tianjin University of Traditional Chinese Medicine, Tianjin, China; ^2^ Institute of Traditional Chinese Medicine, Tianjin University of Traditional Chinese Medicine, Tianjin, China; ^3^ Department of Biochemistry and Molecular Biology, School of Basic Medical Sciences, Tianjin Medical University, Tianjin, China

**Keywords:** non-alcoholic fatty liver disease, mitochondrial dysfunction, natural products, oxidative stress, metabolic syndrome

## Abstract

Non-alcoholic fatty liver disease (NAFLD) has become one of the most common chronic liver diseases worldwide, and its prevalence is still growing rapidly. However, the efficient therapies for this liver disease are still limited. Mitochondrial dysfunction has been proven to be closely associated with NAFLD. The mitochondrial injury caused reactive oxygen species (ROS) production, and oxidative stress can aggravate the hepatic lipid accumulation, inflammation, and fibrosis. which contribute to the pathogenesis and progression of NAFLD. Therefore, pharmacological therapies that target mitochondria could be a promising way for the NAFLD intervention. Recently, natural products targeting mitochondria have been extensively studied and have shown promising pharmacological activity. In this review, the recent research progress on therapeutic effects of natural-product-derived compounds that target mitochondria and combat NAFLD was summarized, aiming to provide new potential therapeutic lead compounds and reference for the innovative drug development and clinical treatment of NAFLD.

## Introduction

With the increasing prevalence of obesity, type 2 diabetes, and metabolic syndrome, non-alcoholic fatty liver disease (NAFLD) has become one of the most common chronic liver metabolic diseases (Adams et al., 2005; Zhao et al., 2020). NAFLD is generally characterized by excessive accumulation of stored energy in liver fat that exceeds the ATP requirements of hepatocytes. Excess accumulation of toxic lipids causes oxidative stress and inflammation, leading to damage and death of hepatocytes (Nagalekshmi et al., 2011). It is estimated that approximately 25% of the global population are affected by NAFLD, and its prevalence is still growing rapidly (Younossi et al., 2016; Huang et al., 2020).

NAFLD is the early stage of many more severe hepatic metabolic diseases including a wide spectrum of disorders ranging from non-alcoholic steatohepatitis (NASH) and liver fibrosis to cirrhosis and even hepatocellular carcinoma (HCC) (Adams et al., 2005; White et al., 2012). Insulin resistance (IR) is believed to play a causative role in the pathogenesis of NAFLD. Overnutrition-induced insulin resistance could sensitize hepatocytes to mitochondrial dysfunction and oxidative damage, leading to the increased inflammation and hepatic stellate cells (HSC) activation, which promote the development of advanced forms of liver injuries (Tilg and Moschen, 2010; Nassir and Ibdah, 2014). In addition to the liver disease, NAFLD is also strongly associated with the cardiovascular disease morbidity and mortality. Indeed, patients with NAFLD are more prone to the development of cardiovascular disease (Adams et al., 2017).

Recently, hepatic mitochondrial dysfunction has been implicated in exerting an important role in the pathophysiology of NAFLD. Mitochondrial structure and function alteration are observed in patients with metabolic syndrome and profoundly induce the metabolic disturbances that contribute to the development of NAFLD (Einer et al., 2018). In this review, we discuss the role of mitochondrial dysfunction in NAFLD and focus on the potential therapeutic effect of natural products on NAFLD, and the association between the therapeutic mechanism and mitochondrial function.

### Mitochondria in Non-Alcoholic Fatty Liver Disease

#### Mitochondrial Homeostasis

The liver plays a predominant role in regulating energy metabolism. While it is relatively small in volume compared with the whole body, the proportion of liver cell respiration is much higher. Under the physiological condition, 15% of the organismal oxygen is consumed by the liver, suggesting that hepatocytes are rich in mitochondria, which consume oxygen to produce ATP (Shum et al., 2020). Indeed, mitochondria take up to 18% of the whole hepatocyte volume and exert a key role in the energy generation from nutrient (carbohydrates, lipids, and proteins) oxidation (Di Ciaula et al., 2021). Therefore, mitochondria are extremely important to maintain the normal metabolic function of the liver.

Mitochondria are highly dynamic organelles, which act as the dynamic hub of the cellular energy metabolism network. There are hundreds of enzymes in mitochondrial matrix that are responsible for pyruvate, fatty acids, and citric acid catabolism (Di Ciaula et al., 2021). In physiological condition, glucose is metabolized into pyruvate through glycolysis and then to acetyl-CoA, which is subsequently oxidated to generate ATP by the TCA cycle and oxidative phosphorylation in the hepatocyte mitochondria. In addition, mitochondria can oxidize the fatty acids and amino acids to produce ATP and ketones or urea for gluconeogenesis during the fasting states (Shum et al., 2020). In addition to the energy production, mitochondria also provide the carbon intermediates for anabolic reactions, such as lipid biosynthesis. Specifically, mitochondrion-derived citrate is converted into acetyl-CoA in the hepatocyte cytosol, where acetyl-CoA is consumed to synthesize fatty acids (Wellen and Thompson, 2012). Moreover, beyond nutrients metabolism, mitochondria can also regulate the concentration of cytoplasmic calcium ions and cellular redox status (Zhao et al., 2020). It also involved in the programmed cell death and innate immunity process, in which mitochondria provide energy and the signaling molecule that are needed to communicate with other organelles such as the endoplasmic reticulum and lysosome (Martucciello et al., 2020). Therefore, maintaining mitochondrial homeostasis is particularly important for the balance of cellular physiological and pathological process in the liver.

**FIGURE 1:**
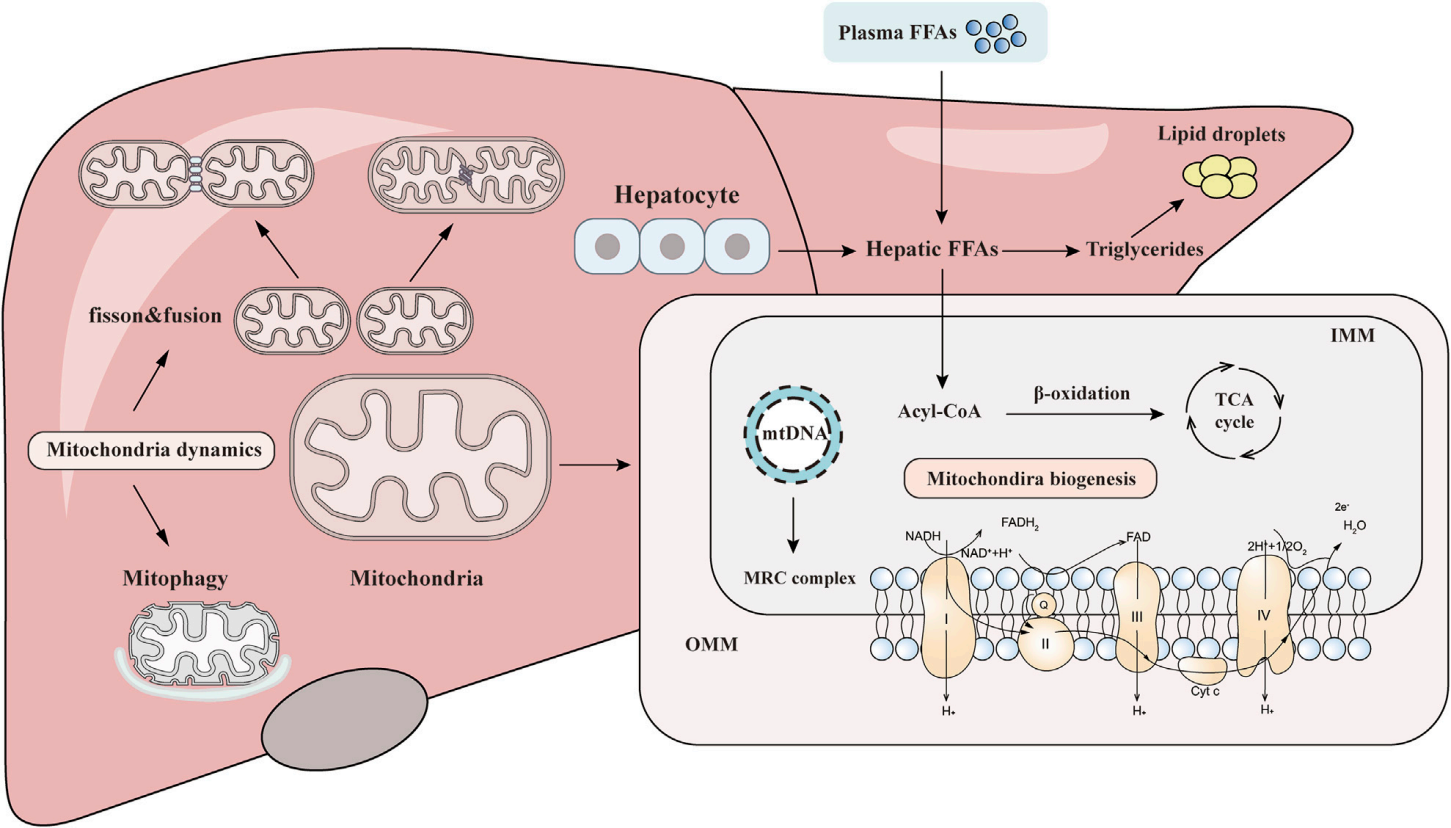
Overview of the association between mitochondrial homeostasis and NAFLD. Mitochondria are dynamic and complex organelles, and their homeostasis is mainly maintained by the balance of mitochondrial biogenesis, mitochondrial fission/fusion, and mitophagy. Mitochondrial injury can aggravate the hepatic lipid accumulation and ROS production and induce the inflammation and fibrosis that contribute to the pathogenesis and progression of NAFLD.

The homeostasis of mitochondria is mainly maintained by the balance of mitochondrial biogenesis, mitochondrial fission/fusion, and mitochondrial autophagy. Once the homeostasis is maladjusted, a certain degree of mitochondrial accumulation will lead to the metabolic disorders of the liver and other tissues (Figure 1).

Mitochondrial biogenesis plays a key role in cellular homeostasis and survival, including mitochondrial DNA (mtDNA) replication, transcription of mtDNA- and nuclear-coding genes, translation, membrane recruitment, protein introduction, and assembly of the OXPHOS complex (Zhu et al., 2014). The process is tightly regulated by a suite of transcription factors, including nuclear respiratory factors (NRF1 and NRF2), estrogen-related receptors (ERRs), and the peroxisome proliferator-activated receptor gamma co-activator 1α (PGC-1α), among which PGC-1α is considered as the master regulator of mitochondrial biogenesis. It can orchestrate the activation of NRFs and ERRs, which therefore regulated two major mitochondrial proliferation involving factors, namely, mitochondrial transcription factor A (TFAM) and transcription factor B proteins (TFBs) (Dominy and Puigserver, 2013; Zhu et al., 2014). Indeed, expression of PGC-1α could promote the mitochondrial proliferation and improve the mitochondrial respiration in mitochondrial defect diseases (Viscomi et al., 2011). Additionally, AMP-activated protein kinase (AMPK) is also involved in the regulation of mitochondrial biogenesis through phosphorylation of PGC-1α (Birkenfeld et al., 2011).

Mitochondrial dynamics includes fusion, fission, and mitophagy, which are another three aspects that modulate the mitochondrial homeostasis. The constant fission and fusion can reshape the mitochondria and repair the damaged components, while the redundant fission or severely damaged mitochondria will be degraded through mitophagy, a mitochondria-specific autophagy, which is the basic process of selective isolation and degradation of damaged mitochondria to maintain the functional integrity of the mitochondrial network and cellular homeostasis. This process is highly regulated by the PTEN-induced kinase 1 (PINK1)–Parkin pathway. Through mitophagy, a cell can avoid excessive reactive oxygen species (ROS) production caused by damaged mitochondria and ensure redox homeostasis. In addition, the mitophagy process can also promote the decomposition of lipid droplets and release free fatty acids transported to intact mitochondria for β-oxidation and increase the energy release (Arroyave-Ospina et al., 2021).

#### Mitochondrial Dysfunction and Non-alcoholic Fatty Liver Disease

Mitochondrial dysfunction is mainly characterized by ROS excessive production, oxidative stress, and respiratory chain reduction. These effects are closely associated with lipid accumulation, inflammation, and hepatic cell death in the NAFLD development. Thus, NAFLD is also considered as a type of mitochondrial disorder (Pessayre and Fromenty, 2005). Under physiological condition, normal mitochondrial fatty acid oxidation (FAO) can support the ATP synthesis and energy supply with controlled superoxide generation. However, when excessive free fatty acids (FFAs) accumulate in cells that cannot be sufficiently handled by mitochondria, the superfluous FFAs will be converted into triglycerides causing the lipid overdecomposition in the liver and leading to steatosis (Pessayre and Fromenty, 2005). In addition, the imbalanced hepatocyte oxidative capacity will make the mitochondria produce ROS greater than the detoxification ability of cellular antioxidants, and these excessive ROS can potentiate oxidative stress through inducing protein oxidation and lipid peroxidation of mitochondrial membranes, impairing respiratory chain activity and causing mtDNA damage (Pessayre, 2007; Besse-Patin et al., 2017), which will further contribute to the mitochondrial dysfunction. Therefore, as early stage of NAFLD (steatosis) progresses to NASH, the impaired mitochondria are becoming insufficient to protect the liver from lipotoxicity due to the continuous FFAs deposition and oxidative damage (Sunny et al., 2017). Additionally, the excessive ROS production may also increase the mitochondrial permeability transition (MPT) pore opening and promote the release of cytochrome C and other proapoptotic factors into the cytosol, causing the hepatocyte death and NASH progression (Haouzi et al., 2000; Ricchelli et al., 2011). Apart from mitochondrial dysfunction, long-term oxidative stress also triggers the inflammation-related signaling pathways activation, such as c-JUN N-terminal kinase (JNK) and nuclear factor kappa B (NF-κB), causing cell inflammatory cytokines release, inflammatory cell infiltration, or even parenchymal hepatic cell death (Pessayre and Fromenty, 2005). For instance, the increased TNF-α can induce the mitochondrial lipid peroxidation and the activation of membrane permeability transition and its subsequent cytochrome C release, resulting in the hepatocyte apoptosis or necrosis, which is considered as a key event in NASH progression (Pessayre et al., 2001). Moreover, the mtDNA released from oxidative damaged liver cells can activate NOD-like receptor family pyrin domain contain 3 (NLRP3) inflammasome and toll-like receptor 9 (TLR9)-mediated inflammatory response and further promote the transition to NASH (Garcia-Martinez et al., 2016; Yang et al., 2020a). Besides, there is strong evidence indicating that ROS and lipid peroxidation can also induce the transforming growth factor beta (TGF-β) production in Kupffer cell and activate the hepatic stellate cells into collagen-producing myofibroblasts, leading to the hepatic fibrosis or even liver cirrhosis (Fromenty et al., 2004).

Patients with more severe NAFLD, such as NASH, are also more prone to mitochondria ultrastructural changes and imbalance of mitochondrial dynamics. Cells failing to remove the damaged mitochondria may cause a large number of damaged mitochondria accumulation and further decrease the ability of the liver to restore its normal function, eventually leading to the cell death and development of advanced NAFLD (Wang et al., 2015). Since mitochondrial autophagy is the major process that is responsible for the clearance of surplus or damaged mitochondria, mitophagy has played a key role in amending NAFLD. Indeed, mitophagy disorders have been found in the livers of both NAFLD patients and mice. Studies have shown that PINK1 or Parkin deficiency leads to defective mitochondrial phagocytosis and exacerbation of NAFLD (Edmunds et al., 2020). In addition, inflammation-induced inactivation of mitofusion2 (Mfn2) activity could also impair mitophagy by decreasing the formation of autophagosome and aggravate hepatic steatosis, resulting in the acceleration of the progression of NASH (Hernández-Alvarez et al., 2019).

Therefore, the mitochondria function and antioxidant status of the liver are crucial in the pathophysiological development of NAFLD. When mitochondria-derived oxidative stress increases, Kelch-like ECH-associated protein 1 (KEAP1) will release nuclear factor (erythrocyte derived 2)-like 2 (Nrf2) and promote its nuclear translocation (Xu et al., 2019a). Activated Nrf2 induces its downstream targets, including nicotinamide adenine dinucleotide phosphate (NADPH) oxidase, quinone oxidoreductase 1 (NQO1), heme oxygenase 1 (HO-1), superoxide dismutase (SOD), catalase, and γ-glutamate cysteine ligase (GCL) expression and increase the antioxidant capacity of the hepatic cell. Correspondingly, Nrf2 deficiency affects the activity of mitochondrial complex I and increase the production of ROS, while Nrf2-deficient mice with fatty liver phenotype can be reversed by increasing the expression of liver antioxidant genes and modulation of lipid metabolism-related genes such as PPARα and SREBP1c (Arroyave-Ospina et al., 2021).

### Application of Natural Products in Non-alcoholic Fatty Liver Disease

The current therapy for NAFLD include diet change, exercise, and pharmacological intervention. However, there are still no specific drug that has been approved for clinical use. Medications for other conditions are often used to relieve NAFLD symptoms, for example, insulin sensitization agents (pioglitazone), lipid-lowering agents (statins), cholesterol absorption inhibitors (ezetimibe), antioxidants (vitamin E), weight loss agents (orlistat), and intestinal probiotics (Kikuzaki et al., 1996; Li et al., 2005; Geng et al., 2011). Studies have shown that regulating lipid metabolism, oxidation, and inflammation-related targets can affect the occurrence and development of NAFLD (Kibble et al., 2015). Especially, the mitochondrial targeting therapy may be one of the effective options to ameliorate liver injury. It has been proven that activation of mitochondrial enzymes, such as PPARα, by specific agonists could markedly increase the lipid metabolism and inhibit the development of hepatic steatosis (Arroyave-Ospina et al., 2021). Vitamin E, a lipophilic antioxidant, has been widely used to treat the patients with NAFLD and NASH (Ji et al., 2014). High dose of vitamin E can effectively improve the steatosis and alleviate the liver injury in NASH patients (Chalasani et al., 2018). Thus, antioxidants targeting mitochondria appear to be a valid strategy for treating NAFLD.

**FIGURE 2:**
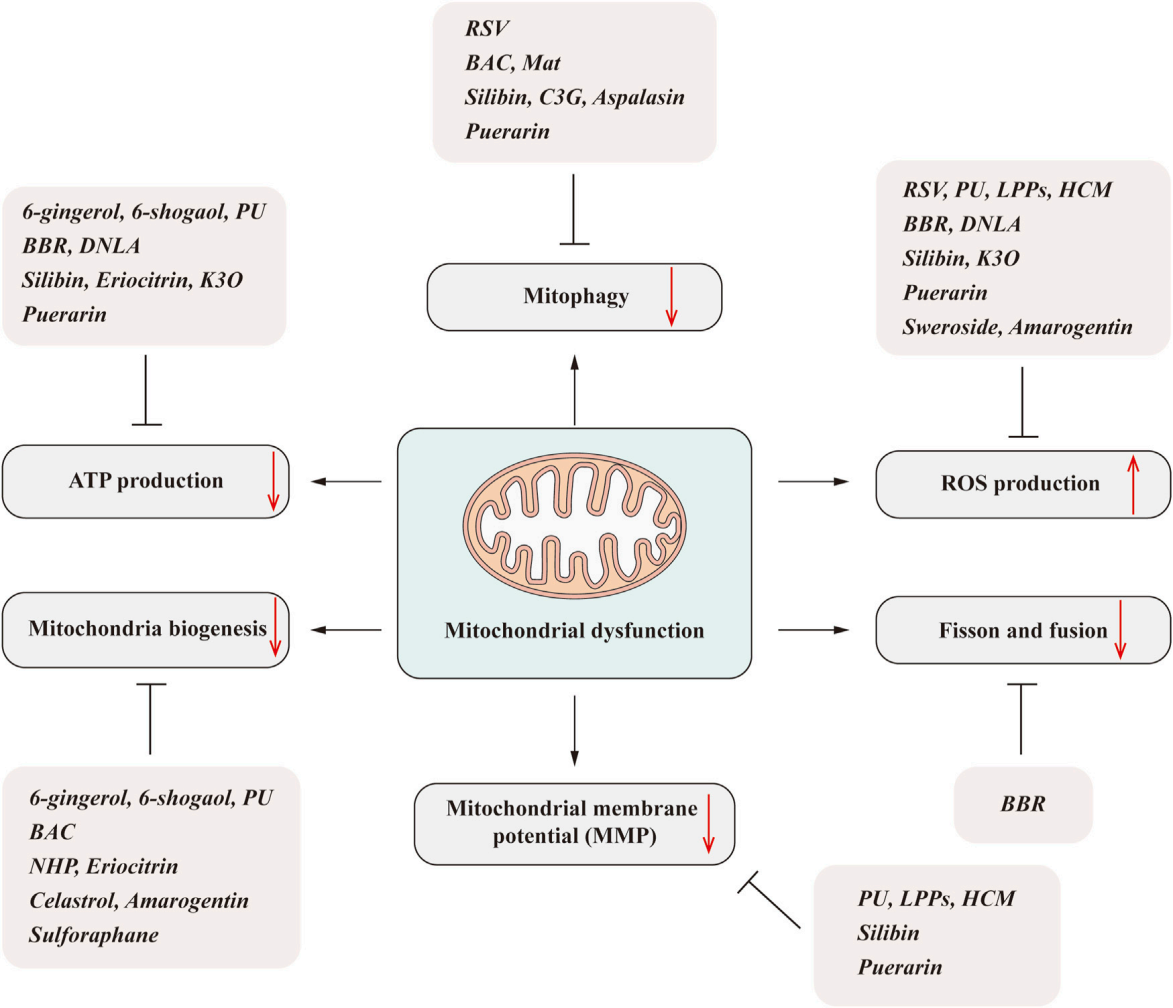
Natural products ameliorate NAFLD by regulating mitochondrial dysfunction. Mitochondrial dysfunction includes many aspects, such as the decreased ATP generation, mitochondrial biogenesis reduction, impaired mitophagy, imbalanced fission/fusion, and increased ROS production. The natural products, including phenols, alkaloids, flavonoids, isoflavones, and terpenoids, can significantly improve one or several aspects that linked to the mitochondrial dysfunction and subsequently improve the NAFLD.

Over the years, many medicinal plants derived from nature have been developed, which are also important sources of many biological compounds. Due to the special structure and molecular diversity of natural products, their activities have been extensively investigated and their important pharmacological role in anti-inflammation, anti-oxidation, and liver protection revealed (Al-Hrout et al., 2018; Hamza et al., 2018; Al-Dabbagh et al., 2019). Recently, accumulating evidence has suggested that natural products can increase mitochondrial function and further improve its associated metabolic diseases, including fatty liver disease such as NAFLD, diabetes, and diabetic complications (Lee et al., 2020). Due to the low toxicity and side effects of natural medicines, it has become a complementary option for the prevention and treatment of NAFLD (El-Kharrag et al., 2017). It is estimated that 40% of Food and Drug Administration (FDA)-approved treatments are natural ingredients or derivatives (El-Kharrag et al., 2017; Al-Hrout et al., 2018). Among the natural products, terpenoids, such as tripterine and triptolide, phenolic compound curcumin, and terpenoid berberine all have good anti-inflammatory and antioxidant activities, suggesting their application prospects in the treatment of mitochondrial dysfunction-related liver disease (Figure 2).

### Phenolics

Resveratrol (RSV), a polyphenolic organic compound, can be extracted from grapes and other plants. RSV has pharmacological effects such as inhibiting adipogenesis and promoting mitochondrial biogenesis to enhance mitochondrial activity (Kim et al., 2014). Studies have shown that resveratrol (20 μM) can inhibit glucose-induced steatosis in HepG2 cells and improve its mitochondrial activity without affecting the cell viability, suggesting the beneficial role of RSV in the treatment of mitochondrial dysfunction (Izdebska et al., 2018). Indeed, RSV can reduce HFD-induced high triglyceride and restore the core component of mitochondrial electron transport chain gene expression, such as COQs. Importantly, the levels and ratios of PINK1 and Parkin are also affected by the RSV intervention and therefore affecting mitochondrial dynamics and mitophagy (Meza-Torres et al., 2020). In addition, RSV can also improve OA-induced lipid accumulation and mitochondrial dysfunction in HepG2 cells and increase mitochondrial membrane potential (MMP) and the expression levels of Sirt1, PPARγ and PGC-1α, thus promoting mitochondrial biogenesis (Rafiei et al., 2019). A recent study also suggests that RSV supplementation in HFD diet-fed rodents could markedly induce hepatic uncoupling protein 2 (UCP2) expression, increase mitochondrial numbers, and inhibit inflammatory responses (Poulsen et al., 2012). In the randomized double-blind crossover study, subjects treated with 150 mg resveratrol for 30 days could significantly reduce the hepatic lipid content, serum triglyceride (TG), alanine aminotransferase (ALT), and inflammatory markers. Furthermore, resveratrol also improved the *ex vivo* mitochondrial function (Timmers et al., 2011; Timmers et al., 2016).

As a folk medicine, pomegranate fruit has been used to treat various diseases. Currently, pomegranate juice and its derivatives are widely used for health promotion (Cerdá et al., 2003). The addition of pomegranate extract (PE) effectively reduced ATP consumption and downregulated the expression of hepatic UCP2 in SD rats, thus avoiding the possibility of ATP depletion (Zou et al., 2014; Hou et al., 2019).

Of the polyphenols found in PE, punicalagin (PU) is the most abundant ellagic tannin. It has been shown to exert antioxidant and anti-inflammatory biological activity (Heber, 2008; Chen et al., 2012) and plays a regulatory role in HFD-induced obesity, insulin resistance, and NAFLD. PU treatment ameliorated palmitate-induced mitochondrial membrane potential lost, ATP depletion, and ROS production, while it increased the mitochondrial complex activities, mtDNA copy number, and mitochondrial fusion-related proteins expression in HepG2 cells (Yan et al., 2016; Cao et al., 2020). Thus, PU could protect the mitochondrial function and restore the mitochondrial morphology and further block mitochondria-mediated caspase-dependent apoptosis. In addition, PU also contribute to the elimination of oxidative stress by increasing SOD activity in the liver (Zou et al., 2014; Yan et al., 2016; Cao et al., 2020). These protective effects of PU are closely associated with PGC-1α and Keap1-Nrf2 signaling pathway activation, suggesting that PU might be a potential supplementary therapeutic agent for mitochondria dysfunction-related liver diseases.

Mounting evidence suggests that the antioxidant effect of polyphenols may be beneficial for the improvement of hepatic lipid accumulation, liver inflammation, and fibrosis. Litchi pulp phenol (LPP) is a high content of phenolic compounds in litchi, which has antioxidant activity *in vitro* and *in vivo* (Su et al., 2016). Studies have shown that LPPs exert a protective effect on mice liver. After LPPs treatment, the levels of ALT, aspartate aminotransferase (AST), and thiobarbituric acid reactive substance (TBARS) in serum were significantly decreased, while the levels of glutathione (GSH), glutathione peroxidase (GPX), SOD, and catalase (CAT) in the liver were increased. LPPs can increase the activity of mitochondrial respiratory chain complex and Na^+^ K^+^ ATPase and decrease the level of mitochondrial membrane potential and the production of ROS (Su et al., 2016). These indicate that LPPs modulate liver injury through scavenging free radicals and regulating mitochondrial dysfunction. Moreover, it significantly reduced mitochondrial protein oxidation by restoring complex I, II, and IV activities (Xiao et al., 2017).

Procyanidins, mainly found in grapes, cocoa, and green tea, are the most abundant polyphenols in human diet. They exert a variety of biological functions such as antioxidant, anti-inflammatory, and hypolipidemic effects. Grape seed proanthocyanidins extract has been used as a bioactive dietary supplement due to its regulatory function in metabolic disorders (Akaberi and Hosseinzadeh, 2016). Açai seed extract (ASE), rich in procyanidins (88%), shows better therapeutic effect than rosuvastatin in improving oxidative damage under the condition that it has similar effects on liver steatosis and hyperlipidemia (Tavares et al., 2020). In addition, the combination treatment of grape seed procyanidins and metformin can more effectively reduce the hepatic TG levels than metformin in NAFLD (Yogalakshmi et al., 2013). In fact, the content of procyanidins B1, B2, and B3 isolated from proanthocyanidins exhibited excellent antioxidant function (Shimada et al., 2012). Both procyanidin B1 and B3 upregulated the expression levels of PGC-1α, NRF1, and TFAM and improved the mitochondrial biogenesis. Procyanidin B1 can also markedly reduce the expression of Drp1 and increase the expression of Mfn2, while procyanidin B3 induces the expression of Mfn1 and Mfn2, thereby maintaining mitochondrial morphology and function (Tie et al., 2020). Moreover, it is reported that procyanidin B2 can significantly reduce lipid accumulation and excessive ROS production in HepG2 cells. Mechanistically, it inhibits FFA-induced hepatic steatosis and oxidative stress by regulating TFEB-mediated lysosomal pathway and restoring mitochondrial membrane potential (Su et al., 2018).

*Zingiber officinale* Roscoe, commonly known as ginger, exerts a significant role in mitochondrial biogenesis and the lipid metabolism (Deng et al., 2019b). Ginger extract (GE) can promote OXPHOS of liver and activate the AMPK/PGC-1α signaling pathway. GE induces the production of ATP and the activity of mitochondrial respiratory chain complex I and IV, which promotes mitochondrial biogenesis and improves mitochondrial function. Consistently, 6-gingerol and 6-shogaol extracted from ginger could induce the oxygen consumption and intrascapular temperature in mice liver by increasing mtDNA copy number (Deng et al., 2019b).

Helenalin isolated from *Centipeda minima* (L.) A. Braun and Asch. (HCM) has been found to have anti-inflammatory and antioxidative effects. HCM can significantly reduce oxidative stress, lipid peroxidation, and production of ROS by activating the Nrf2 pathway. Thereby, it protects mitochondria function and reduces the liver damage. Additionally, HCM could also significantly reduce the production of inflammatory cytokines by inhibiting Toll-like receptor 4 (TLR4) signal transduction and NF-κB activation, which may further protect the mitochondria from inflammation related injury and subsequently decreasing the hepatocyte apoptosis (Li et al., 2019b).

### Alkaloids

Benzoyl aconitine (BAC) is one of the representative traditional alkaloids in *Fuzi* (*Aconitum carmichaeli* Debeaux). BAC induces mitochondrial biogenesis through the activation of AMPK/PGC-1α signaling cascade. AMPK is a kinase that responds to mitochondrial function by regulating mitochondrial biogenesis and autophagy (Rabinovitch et al., 2017; Foretz et al., 2018). It has been reported that BAC could increase HepG2 cells’ mitochondrial mass and mtDNA copy number in a dose-dependent manner without affecting cell proliferation. As for ATP production, the most important function of mitochondria, BAC can increase its production through promoting the oxygen consumption rate and the expression of OXPHOS-related proteins, including NDUFS1 (Complex I), SDHA (Complex II), UQCRC1 (Complex III), COX4 (Complex IV), and ATP5A1 (Complex V) in HepG2 cells (Deng et al., 2019a). Consistently, BAC protects mice from liver steatosis and inflammation by improving systemic glucose homeostasis, reducing fat mass, and increasing autophagy flux (Garcia et al., 2019).

Matrine (Mat) is a tetracyclo-quinolizidine alkaloid, which is mainly derived from leguminosae such as *Sophora flavescens* Aiton. It has been reported that Mat exerts pharmacological effects on improving liver function in patients with hepatitis (Liang et al., 2019). In addition, studies have shown that Mat and oxymatrine (Oxy-Mat) can inhibit steatohepatitis (Mahzari et al., 2018). Mechanistically, it was suggested that Mat could downregulate the levels of lipogenesis-related proteins, such as sterol regulatory element-binding protein 1c (SREBP-1c), fatty acid synthase (FAS), and acetyl-coa-carboxylase (ACC) both in HFD mice and L02 cells. The decreased fatty acid synthesis will reduce the fatty acid overaccumulation-induced mitochondria injury and improve the hepatic steatosis (Gao et al., 2018). In addition to reducing lipogenesis in the liver, Mat can also significantly reduce the palmitic-acid-induced mitochondrial dysfunction and endoplasmic reticulum stress (ER stress) in L02 cells. These effects are strongly associated with the downregulated level of intracellular calcium, since the abnormal release of ER calcium could lead to both mitochondrial dysfunction and ER stress. Mechanistically, Mat regulates cytosolic calcium homeostasis mainly through its inhibition effect on sarcoplasmic/endoplasmic reticulum calcium ATPase (SERCA) pump (Gao et al., 2018). Moreover, it is also reported that Mat treatment can enhance the mitophagy and alleviate the mitochondrial-damage-associated oxidative stress (Wang et al., 2019a).

*Dendrobium nobile* Lindl. alkaloids (DNLAs) are the main active ingredients of *D. nobile*, which were found to have a protective effect on hepatic lipid homeostasis and mitochondrial function (Li et al., 2019a). It has been shown that DNLA can combat mitochondrial oxidative stress and reduce its oxidative damage. DNLA treatment can improve oxygen consumption, reverse mitochondrial respiratory depression, and increase ATP production *via* regulating Nrf2 signaling pathway in mice. Consequently, mitochondrial H_2_O_2_ content and malondialdehyde (MDA) production were reduced, while GSH level and Mn-SOD activity were significantly increased with DNLA treatment. Furthermore, DNLAs also decrease the level of membrane-permeable ROS and further inhibit the oxidative damage of mitochondrial lipids (Zhou et al., 2020).

Berberine (BBR) is an isoquinoline alkaloid that can improve glucose metabolism and enhance insulin sensitivity. In addition, BBR also has pharmacological effects on reducing body weight, cholesterol, and triglyceride levels (Kong et al., 2008). As a promising drug for metabolism disorders, BBR can effectively improve mitochondrial swelling and promote mitochondrial fusion (Yu et al., 2021). It has been reported that silent mating-type information regulation 2 homolog 3 (SIRT3) can regulate the mitochondrial β-oxidation through deacetylating long-chain acyl-coenzyme A dehydrogenase (LCAD). In both high-fat fed mice and rats, BBR intervention can notably promote the SIRT3 expression and activation, thereby improving systematic and inhibiting the progression of hepatic steatosis (Teodoro et al., 2013; Xu et al., 2019b). Besides, BBR also markedly reduced Nox2-dependent mitochondrial ROS production and improve non-esterified fatty acid impaired mitochondrial respiratory chain function by regulating Nrf2 signaling and PGC-1α expression (Sun et al., 2017; Shi et al., 2018). Recently, a randomized controlled trial demonstrated that berberine ursodeoxycholate has a broad spectrum of metabolic activity in patients with NASH and diabetes. It can reduce the liver lipid content with apparent improvement in hepatic inflammation and injury. Importantly, it is relatively well tolerated with oral administration, suggesting that berberine ursodeoxycholate could be a feasibility way for the combined treatment of NASH and diabetes (Harrison et al., 2021).

### Flavonoids

Flavonoids are commonly found in vegetables, fruits, tea, coffee, and other drinks in daily life. Currently, they have shown wide range of biological pharmacological activities, such as antibacterial, anti-inflammatory, and ROS clearance (Martucciello et al., 2020).

Silymarin (SM), extracted from the Mediterranean plant *Silybum marianum* (L.) Gaertn, contains various flavonolignans. Silymarin is reported to optimize the electron transport chain under oxidative stress, maintaining the integrity of the mitochondrial respiratory chain, thereby reducing electron leakage and directly reducing the activity of ROS-producing enzymes in the mitochondria (Surai, 2015; Baldini et al., 2020). In randomized trial, Silymarin remarkably attenuates the NAFLD activity score (NAS) and fibrosis score in patients with NASH, indicating that Silymarin can be a promising phytotherapy for NAFLD and NASH patients (Wah Kheong et al., 2017; Zhong et al., 2017). As the main component of silymarin, silybin is found to eliminate ROS, reduce lipid peroxidation, inhibit apoptosis, and reduce oxidative stress in HepG2 cells (Esselun et al., 2019). In addition, Silybin can reduce the activation of oxidative-stress-dependent transcription factor NF-κB and promote autophagy by restoring aquaporin 9 (AQP9) and glycerol permeability levels.

Recently, silybin-phospholipid (SILIPHOS), an antioxidant complex, has shown hepatoprotective and antifibrosis effects in rat NASH model. It regulates mitochondrial energy metabolism by preventing proton leakage. Additionally, it can also reduce glutathione consumption and mitochondrial H_2_O_2_ production and restore the decreased ATP synthesis caused by chronic liver disease (Serviddio et al., 2010). Realsil is another silybin complex containing vitamin E, which greatly enhances the bioavailability of silymarin and also show antioxidant effects against mitochondrial ROS and NO production (Grattagliano, 2013). In NAFLD and NASH patients, Realsil treatment could significantly reduce the serum lipid peroxidation and NAS score and restore antioxidant capacity of hepatocytes (Stiuso et al., 2014; Federico et al., 2020).

Anthocyanins are one of the main color substances in plant flowers and fruits and known as the seventh most essential nutrient for the human body. Mulberry anthocyanins have been widely used in the fields of food and health products due to their high extraction rate and stable structure. Anthocyanins extracted from bilberry and blackcurrant have been reported to enhance the activation of AMPK/PCC-1α signaling pathway, which in turn protects mitochondrial biogenesis and electron transport chain in NASH mice (Tang et al., 2015). Cyanidin-3-O-glucoside (C3G) is one of the main components of anthocyanin in mulberry, which has been shown to reduce visceral fat and weight gain in obese adults and rats (Li et al., 2020). During the onset of NAFLD, if a large number of damaged mitochondria accumulate, ROS excessive production and mitochondrial autophagy damage may lead to a second hit in the development of NAFLD, causing more serious liver disease. It was reported that C3G can inhibit liver oxidative stress, NLRP3 inflammasome activation, and steatosis in mice and NAFLD patients. In high-fat diet fed mice, C3G treatment increases LC3-II protein abundance, autophagosomes number, and mitochondrial localization, promoting PINK1/Parkin-mediated mitophagy to clear the damaged mitochondria [33]. Thus, the lipid droplets are induced to decompose *via* lipophagy (McWilliams and Muqit, 2017; Schulze et al., 2017), thereby promoting more free fatty acids release into mitochondria for β-oxidation to alleviate hepatic lipid accumulation. At the same time, the damaged mitochondria are also decomposed through mitochondrial autophagy, which further reduces oxidative stress to maintain mitochondrial homeostasis [33].

A growing number of epidemiological and clinical studies have shown that citrus fruits, such as *Citrus* × *aurantium* L. (*C. aurantium*), exert a positive effect on glucose and lipid metabolism. Neohesperidin (NHP), as one of the most abundant flavonoids in *C. aurantium*, is reported to enhance mitochondrial biogenesis in HFD mice by activating the AMPK/PGC-1α signaling pathway (Wang et al., 2020). After NHP intervention, the mice hepatic lipid accumulation and the liver steatosis were significantly improved. Indeed, NHP also promotes fatty acid oxidation, reduces insulin resistance, and improves glucose homeostasis in HFD mice.

Among all the bioactive molecules in lemons, eriocitrin is the major flavonoid with antioxidant activity that can decrease lipid levels and reduce oxidative stress without causing toxicological manifestations. Eriocitrin can significantly upregulate the mRNA levels of ACOX1 and ACADM. More importantly, it promotes mitochondrial β-oxidation and biogenesis and ameliorates HFD-induced hepatic steatosis (Hiramitsu et al., 2015) in palmitate-induced HepG2 cells. After treatment with eriocitrin, mtDNA content was significantly increased in a dose-dependent manner, accompanied with increased intracellular ATP production. These suggests that eriocitrin may increase mitochondrial biogenesis and restore the activity of respiratory chain complex, thereby restoring the mitochondrial function.

Kaempferol-3-O-glucuronide (K3O) is a natural chemical component extracted from Holly plants. It has antioxidant, lipid metabolism regulation, and anti-inflammatory effects. Studies have shown that K3O can reduce the oxidative stress and lipid peroxidation in the liver, reduce hepatic steatosis, and alleviate NAFLD. K3O treatment decreases H_2_O_2_-induced ROS production in HepG2 cells. Additionally, it can also significantly reduce MDA and increase the level of GSH-Px. In the mechanism study, the results suggest that the protective effect of K3O is associated with Nrf2/Keap1 signaling activation (Deng et al., 2021).

In addition to activating mitochondrial biogenesis, AMPK can also improve mitochondrial function by promoting mitochondrial autophagosome formation and increasing autophagy flux. Aspalasin is a rooibos flavonoid that can activate AMPK signaling pathway to increase hepatic energy expenditure and improve liver lipid metabolism. In palmitate-induced liver cells, impaired hepatic substrate metabolism together with defective insulin signaling pathway is strongly associated with decreased Akt protein expression and mitochondrial respiratory rate (Mazibuko-Mbeje et al., 2019). However, aspalasin administration significantly improve mitochondrial dysfunction through promoting the respiration and ATP production. These results suggest that aspalasin could protect from mitochondria-related liver injury by regulating AMPK signaling, subsequently inhibiting the steatosis development.

### Isoflavone

Puerarin is isolated from the roots of *Pueraria lobata* (Wild.) Ohwi. As an isoflavone compound, it has shown the biological activity in restoring mitochondrial dysfunction, preventing oxidative stress and inflammation, and improving lipid/glucose metabolism. In high-fat high-sucrose (HFHS) diet fed mice, puerarin has a certain therapeutic potential to inhibit NAFLD. It can increase the mitochondrial membrane potential and ATP generation, therefore improving liver mitochondrial functional homeostasis and decreasing the ROS production. It suggested that the effects of puerarin are closely related to its role in NAD^+^ replenishing and AMPK activation (HOU et al., 2020; Wang et al., 2019b). Apart from mitochondrial function protection, puerarin is also involved in regulating mitochondrial dynamics. It can modulate the mitochondrial fission and fusion by increasing the Mfn2 and Opa1 expression and decreasing Drp1 expression. Moreover, palmitate-impaired mitophagy is also restored by puerarin *via* increasing PINK1/Parkin expression (Chen et al., 2018) Thus, these results indicate that puerarin could be a potential therapeutic agent for the treatment of NAFLD.

### Terpenoids

Celastrol is a potent anti-inflammatory pentacyclic triterpene extracted from the root of *Tripterygium wilfordii* Hook. f. It has been reported that rich accumulation of palmitic acid from the diet can lead to elevated levels of diacylglycerol (DAGS) and ceramides (Carta et al., 2017). These accumulated toxic lipids can induce lipid toxicity, leading to mitochondrial dysfunction and endoplasmic reticulum stress (Arroyave-Ospina et al., 2021). It has been reported that celastrol can improve mitochondrial dysfunction and insulin sensitivity through reducing mitochondrial oxidative stress and thus enhance fatty acid oxidation in palmitic-acid-induced C3A human hepatocytes (Abu et al., 2017). Importantly, celastrol can stimulate mitochondrial biogenesis and increase cell antioxidant capacity by activating AMPK-SIRT1 signaling pathways, which is associated with increased SIRT1 deacetylation activity and activation of PGC-1α and coactivation of nuclear respiratory factor 1 (NRF1) expression (Abu et al., 2020). In addition, Hu et al. also found that celastrol could promote Nur77, a nuclear receptor, translocation from the nucleus into mitochondria. Nur77 interacted with tumor necrosis factor receptor-associated factor 2 (TRAF2) and p62 to facilitate the injured mitochondria clearance by mitophagy (Hu et al., 2017). Therefore, celastrol might be a good candidate therapeutic agent for the treatment of mitochondrial dysfunction and inflammation-triggered NASH.

Sweroside is the secoiridoid extracted from *Swertia pseudochinensis* H. Hara. A recent study demonstrated that sweroside is a hepatoprotective agent against NAFLD. Mice treated with sweroside were resistant to HFD-induced weight gain, insulin resistance, and hepatic steatosis. Sweroside can reduce the number of lipid droplets and inflammatory cell infiltration in the liver. These beneficial effects of sweroside are closely related to its role in regulating PPARα and CD36 and FGF21 expression (Yang et al., 2020b). There is evidence that NLRP3 inflammasome is increased during the occurrence and development of NAFLD (Mridha et al., 2017; Yang et al., 2020a). Mitochondria-injury-related ROS release can induce NLRP3 inflammasome activation-mediated proinflammatory cytokine expression and aggravate the NASH development (Kim et al., 2020; Wu et al., 2020). Sweroside has been reported to alleviate oxidative stress and intercellular ROS, which can therefore inhibit the activation of NLRP3 inflammasome, reduce the levels of IL-1β, and subsequently reduce inflammation and further improve metabolic diseases (Ma et al., 2018). These results suggest that sweroside may be a good therapeutic agent for NAFLD and NASH treatment.

Gentianaceae plant extracts have been widely used in food additives, tea, or medicine to treat various human diseases and disorders (Dai et al., 2018). Amarogentin, a gentian iridoid, has been shown to have a protective effect on liver injury and improve mitochondrial dysfunction by regulating the expression level of liver CYP450 system. When mitochondrial function is impaired, the expression level of mtDNA-encoded genes will change, which will eventually lead to the impaired energy production and liver failure (El-Hattab and Scaglia, 2013). Amarogentin can reverse mtDNA damage, significantly reduce mtDNA deletion in HepG2 cells, and prevent cell apoptosis. In addition, amarogentin could also reduce the production of ROS and increase antioxidant capacity in HepG2 cells, thereby improving mitochondrial damage (Dai et al., 2018).

### Other Compounds

*Polygonatum kingianum* Collett Hemsl. (PK) has been used as herb and nutritional ingredient for centuries. The main active components in PK can be isolated as polysaccharides, steroid saponins, triterpenoid saponins, and isoflavones (Zhao et al., 2018). PK has the pharmacological activity of regulating lipid metabolism and promoting mitochondrial function (Yang et al., 2019), and it has been used to treat mitochondrial dysfunction and alleviate NAFLD. The reduction in HFD-induced NAFLD by PK is related to the improvement of mitochondrial antioxidant status, energy metabolism, and β-oxidation of fatty acids. In addition, PK can also inhibit the apoptosis of liver cells. PK extract administration can restore the activities of GSH-Px, SOD, Na^+^-K^+^-ATPase, and complex I and II in SD rats and reduce the content of MDA in liver mitochondria. It can also markedly upregulate the expression of CPT-1α mRNA and downregulate the expression of UCP-2, thereby regulating mitochondrial biogenesis and fatty acid metabolism. In addition, PK can increase the expression of Bcl-2 in liver cells and inhibit cytochrome C release from mitochondria and subsequent apoptosis-related proteins expression.

Sulforaphane is an isothiocyanate extracted from cruciferous vegetables, best derived from broccoli buds, and most of its activity is attributed to its ability to regulate the signaling pathway of Keap1-Nrf2-antioxidant reaction element (Kubo et al., 2017). Sulforaphane and its precursor glucoraphanin have been considered as the most effective natural inducers of Nrf2. It is reported that glucoraphanin can effectively inhibit the HFD-fed mice weight gain and reduce liver steatosis through improving lipid peroxidation. In addition, it also increases the energy expenditure and mitochondrial uncoupled protein 1 (UCP1) level and therefore suppress the development of NASH (Nagata et al., 2017). Oxidative stress and calcium ion can induce the mitochondrial permeability transition pore (mPTP) opening and subsequent membrane depolarization, which is often associated with cell death, while sulforaphane supplement significantly inhibits the redox-induced mPTP opening and protects from mitochondrial dysfunction in rats (Greco et al., 2011). Furthermore, sulforaphane ameliorates the mitochondrial swelling and promotes the mitochondrial biogenesis through regulating PGC-1α-dependent pathway (Lei et al., 2019). Taken together, sulforaphane and glucoraphanin exert pharmacological activity of improving mitochondrial function and inhibiting the development of NAFLD.

## Discussion

As the prevalence of NAFLD increases over the decades, it has become one of the most common chronic liver diseases worldwide. However, the efficient therapies for this liver disease are still limited. So far, only physical exercise and dietary modification are recommended by FDA. Recently, mounting evidence has suggested that mitochondrial dysfunction is closely associated with the NAFLD development. Mitochondrial injury can aggravate the hepatic lipid accumulation and ROS production and induce the inflammation and fibrosis that contribute to the pathogenesis and progression of NAFLD. Thus, pharmacological therapies that target mitochondria could be a promising way for the NAFLD intervention in clinics. Indeed, many mitochondrial targeted agents derived from natural products have been extensively studied and have revealed good pharmacological activities in combating the NAFLD (Rafiei et al., 2019).

Most of the natural products that regulate the mitochondria are mainly through promoting mitochondrial function and adjusting the mitochondrial dynamics to alleviate the NAFLD development. Increased lipid flux can lead to insufficient mitochondrial oxidation and aggravate ROS production, leading to oxidative stress. The balance between oxidants and antioxidants plays a key role in maintaining mitochondrial function during the development of NAFLD. Natural products that act as mitochondrial biogenesis inducer and selective antioxidants are essential in improving the mitochondrial homeostasis and reducing oxidative stress and thereby protecting from NAFLD. As summarized in this review, the alkaloids berberine, phenolic compounds 6-gingerol and 6-shogaol, flavonoids NHP, and terpenoids celastrol and amarogentin all showed pharmacological activities to produce functional mitochondria, which can promote mitochondrial biogenesis. These natural antioxidants increase liver antioxidant capacity by improving mitochondrial homeostasis, thus providing effective treatment for chronic metabolic liver diseases such as NAFLD.

Mitophagy affects the lipid phagocytosis process and can eliminate damaged mitochondria. The maintenance of mitochondrial homeostasis is closely related to mitochondrial autophagy. Mitophagy of damaged mitochondria can ensure the normal decomposition and energy release of lipid droplets. Flavonoids C3G and aspalasin can promote mitochondrial autophagy, increase autophagy flux, and reduce liver inflammation and steatosis. In addition to improving mitochondrial biogenesis, celastrol also improved NASH by promoting mitophagy to inhibit inflammatory responses.

Although various pharmacological mitochondria-protection activities have been exhibited by the core components of natural products, the therapeutic utility of some chemicals are partly compromised due to their poor water solubility and low bioavailability. For instance, to achieve therapeutic efficacy, berberine has to be used at relatively large oral doses in mice (Turner et al., 2008), which limits the development and application of berberine as the pharmacological preparation. Therefore, the design and structural modification of these natural chemicals to get eligible derivatives with good pharmacological and pharmacokinetic profile are extremely important for the future development of mitochondrial targeting medicine (Gaba et al., 2021). Recently, combination therapy is considered as a potential therapeutic option for the mitochondria-related liver disorders. This therapeutic strategy includes both backbone and complementary treatment, which can maximize the therapeutic effects of two or more natural products, or even combined with common chemical medicine. Thus, combination therapy may also be a good way to make full use of natural mitochondria medicine.

Herein, we revealed the relationship between mitochondrial function and the pathogenesis of NAFLD and summarized the protective effect of natural products on NAFLD and its subsequent chronic liver diseases by improving mitochondrial homeostasis (Table 1). By developing natural-products-derived compounds that target mitochondria will provide new potential therapeutic approaches and clinical perspective for treating mitochondrial dysfunction and spare the liver from NAFLD.

**TABLE 1:** Mechanisms of natural products and active components in the treatment of NAFLD.

Classification	Natural Product	Animal model	Cell model	Function	Mechanism/Target	Reference
Phenolics	Resveratrol (RSV)	C57BL/6 mice (RSV 20 mg/kg)	HepG2 cells (RSV 1 μM) AML-12 cells	Mitochondrial biogenesis promotion, ROS reduction	eNOS/NO/cGMP pathway, Akt/Nrf2 pathway	Kim et al. (2014)
		—	HepG2 cells (RSV 10, 20 μM)	Mitochondrial dynamic and β-oxidation promotion	FAS, *p*-AMPK, CPT1α, Sirt1, PPARγ, SREBP-1c	Izdebska et al. (2018); Rafiei et al. (2019)
		C57BL/6J mice	—	Mitophagy promotion	PINK1/Parkin pathway, CoQs	Meza-Torres et al. (2020)
		Wistar rats (RSV 100 mg)	—	Mitochondrial number elevation	UCP2	Poulsen et al. (2012)
	Punicalagin (PU)	SD rats (PE 50, 150 mg/kg)	HepG2 cells (4 μg/ml PU and 10 μg/ml PE)	ROS reduction, increase ATP production	Nrf2/HO-1/NQO1 pathway, UCP2, PGC-1α, ACADL, ACADM (MRC complex)	Zou et al. (2014)
		C57BL/6J mice (PU 50, 200 mg/kg)	HepG2 cells (PU 10, 20 μg/ml)	Mitochondrial biogenesis promotion, MMP recovery	Nrf2, PGC-1α, FAS, ACC1	Cao et al. (2020)
		—	HepG2 cells (PU 10, 20 μg/ml)	ROS reduction; mitochondrial translocation reduction	Keap1-Nrf2 pathway	Zou et al. (2014)
	Litchi pulp phenol (LPPs)	Kunming mice (LPP 50, 100 and 200 mg/kg)	—	ROS reduction; MMP recovery	MRC complex, Na^+^ K^+^ ATPase	Su et al. (2016)
	Procyanidins	ICR mice (procyanidins 50, 200 mg/kg)	3T3-L1 cells (flavan-3-ols 0–100 μM)	Mitochondrial biogenesis promotion	PGC-1α, NRF1, TFAM, Mfn1, Mfn2, Drp1	Tie et al. (2020)
		C57BL/6 mice (procyanidins 50, 150 mg/kg))	HepG2 cells (procyanidin B2 10 μg/ml)	ROS reduction	C/EBPα, SREBP-1c, TNFα, TFEB	Su et al. (2018)
	6-gingerol, 6-shogaol	Balb/c mice (GE 1,2 g/kg)	HepG2 cells (6-gingerol 25, 50, 100, 200 μM)	ATP production, OXPHOS and mitochondrial biogenesis promotion	AMPK/PGC1α pathway, MRC complex	Deng et al. (2019b)
	Helenalin (HCM)	C57BL/6 mice (HCM 0.75, 1.5 and 3 mg/kg)	—	ROS reduction; MMP recovery	Nrf2 pathway, NQO1, HO-1, NF-κB	Li et al. (2019b)
Alkaloids	Benzoyl aconitine (BAC)	Balb/c mice (BAC 10 mg/kg)	HepG2 cells (BAC 25, 50, 75 μM)	OXPHOS, mitochondrial biogenesis, and mitophagy promotion	AMPK pathway, NDUFS1, SDHA, UQCRC1, COX4, ATP5A1	Deng et al. (2019a)
	Matrine (Mat)	C57BL/6J mice (Mat 0.5, 2.5, 10 mg/kg)	L02 cells (Mat 200 and 400 μM)	Maintain cytosolic calcium homeostasis, mitophagy protection	SERCA pump, SREBP1c, FAS, ACC	Gao et al. (2018)
	Dendrobium nobile Lindl. (DNLA)	Wild-type and Nrf2^−/−^mice (DNLA 10 mg/kg)	—	ROS reduction; increase ATP production	Nrf2 pathway	Li et al. (2019a); Zhou et al. (2020)
	Berberine (BBR)	C57BL/6J mice (BBR 0.075, 1.4 g/kg)	—	Mitochondrial swelling improvement; mitochondrial fusion promotion	SCD1, FABP1, CD36, CPT1α	Yu et al. (2021)
		C57BL/6 and Sirt3^−/−^ mice	—	Mitochondrial β-oxidation promotion	Sirt3, LCAD	Teodoro et al. (2013)
		SD rats (BBR 100 mg/kg)	—	Increase ATP production; MMP recovery	Sirt3	Xu et al. (2019b)
		SD rats (BBR 300 mg/kg)	Huh7 cells (BBR 5,10 μM)	ROS reduction	Nrf2, MRC complex	Shi et al. (2018)
		Holstein cows	Bovine hepatocyte (BBR 10, 20 μM)	Increase ATP production	AMPK pathway, PGC1α	Sun et al. (2017)
Flavonoids	Silybin (Sil)	—	Rat hepatoma FaO cells (Sil 50 μM)	Mitophagy promotion	miR-122, AQP9, UCP2, NF-κB	Baldini et al. (2020)
	Silybin-phospholipid complex (SILIPHOS)	Wistar rats (Sil or SILIPHOS 0.4 g/kg)	—	ROS reduction	MRC complex, H_2_O_2_	Serviddio et al. (2010); Grattagliano (2013)
				Increase ATP production		
				MMP recovery		
	Cyanidin-3-O-glucoside (C3G)	Over expression and knockdown of PINK1 mice (C3G 0.2%)	AML-12 cells HepG2 cells (C3G 100 μM)	Mitophagy promotion	PINK1/Parkin pathway, NLRP3	Li et al. (2020)
	Neohesperidin (NHP)	C57BL/6 mice (NHP 50 mg/kg)	HepG2 cells (NHP 100 μM)	Mitochondrial biogenesis promotion	AMPK/PGC-1α pathway	Wang et al. (2020)
	Eriocitrin	Zebrafish (32 mg/day)	HepG2 cells (Erio 1, 3, 10 μM)	Mitochondrial β-oxidation and biogenesis, ATP production promotion	MRC complex, ACOX1, ACADM	Hiramitsu et al. (2015)
	Kaempferol-3-O-glucuronide (K3O)	Zebrafish (10, 20, 40 μM)	HepG2 cells (K3O 10, 15, 20 μM)	ROS reduction ATP production promotion	Nrf2/Keap1 pathway	Deng et al. (2021)
	Aspalasin	—	C3A liver cells (Aspalasin 10 μM)	Mitophagy promotion	PI3K/Akt signaling pathway	Mazibuko-Mbeje et al. (2019)
Isoflavone	Puerarin	C57BL/6 mice (100, 200, 400 mg/kg)	HepG2 cells (0.1, 1, 10 μM)	ROS reduction	PINK1/Parkin signaling pathway, PI3K/Akt signaling pathway, AMPK, OPA1, Mfn2	HOU et al. (2020); Wang et al. (2019b); Chen et al. (2018)
				MMP recovery		
				ATP and mitochondrial autophagy production		
Terpenoids	Celastrol	SD rats (Celastrol 1, 3 mg/kg)	C3A human cells (30, 100 nM)	Mitochondrial biogenesis promotion	AMPK and SIRT1 signaling pathways, PPARγ, PGC-1α, NRF1	Abu et al. (2017); Abu et al. (2020)
		C57BL/6 and Nur77^−/−^ mice (Celastrol 0.1 mg/kg)	HepG2, SMMC-7721, QGY-7703 cells (Celastrol 2, 4 μM)	Increase mitochondrial antioxidant activity and biogenesis	Nur77, p62	Hu et al. (2017)
	Sweroside	C57BL/6 mice (Sweroside 5, 30, 60, 120, 240 mg/kg)	Bone marrow-derived macrophages (BMDMs)	ROS reduction	PPARα, CD36, NLRP3 inflammasome, IL-1β	Yang et al. (2020b); Mridha et al. (2017); Yang et al. (2020a)
			Sweroside (25, 50,10 μM)			
	Amarogentin	—	HepG2 cells (Amarogentin 12.5, 25, 50 μM)	ROS reduction	CYP450 system	Dai et al. (2018)
				Mitochondrial biogenesis promotion		
				Reverse mtDNA damage		
Other compounds	Polygonatum kingianum (PK)	SD rats (PK 1, 2, 4 g/kg)	—	Mitochondrial biogenesis promotion	CPT-1α, UCP-2, MRC complex	Yang et al. (2019)
	Sulforaphane	Rats	—	Increase mitochondrial antioxidant defenses and inhibits redox-sensitive PTP opening	Keap1-Nrf2 pathway	Kubo et al. (2017); Nagata et al. (2017); Greco et al. (2011)
		C57BL/6JSlc mice				
		Wistar Rats (SFN 20 mg/kg)	HHL-5 cells (SFN 1, 5, 10 μM)	Mitochondrial biogenesis promotion	Nrf1, TFAM, PGC-1α, ATGL, HSL	Lei et al. (2019)
				Mitochondrial swelling improvement		
